# Layer Anti-Ferromagnetism on Bilayer Honeycomb Lattice

**DOI:** 10.1038/srep05367

**Published:** 2014-06-20

**Authors:** Hong-Shuai Tao, Yao-Hua Chen, Heng-Fu Lin, Hai-Di Liu, Wu-Ming Liu

**Affiliations:** 1 Beijing National Laboratory for Condensed Matter Physics, Institute of Physics, Chinese Academy of Sciences, Beijing 100190, China

## Abstract

Bilayer honeycomb lattice, with inter-layer tunneling energy, has a parabolic dispersion relation, and the inter-layer hopping can cause the charge imbalance between two sublattices. Here, we investigate the metal-insulator and magnetic phase transitions on the strongly correlated bilayer honeycomb lattice by cellular dynamical mean-field theory combined with continuous time quantum Monte Carlo method. The procedures of magnetic spontaneous symmetry breaking on dimer and non-dimer sites are different, causing a novel phase transition between normal anti-ferromagnet and layer anti-ferromagnet. The whole phase diagrams about the magnetism, temperature, interaction and inter-layer hopping are obtained. Finally, we propose an experimental protocol to observe these phenomena in future optical lattice experiments.

The bilayer honeycomb lattice (BHL) has attracted enormous interest in both experimental and theoretical research. Lots of novel phenomena have been found in BHL, for instance, the quantum Hall effect, quantum spin Hall effect, and chiral superconductivity^1^,^2^,^3^,^4^,^5^,^6^,^7^,^8^,^9^,^10^,^11^. However, the real charge and magnetic order induced by Coulomb interaction are still challenges in the strongly correlated BHL^12^,^13^,^14^,^15^,^16^. A quadratic dispersion relation, signed by two touching bands in the corners of Brillouin zone, can be found in BHL, which is driven by the inter-layer hopping. In addition, the spontaneous symmetry can be broken by the dimers when the inter-layer hopping changes. Some amazing phases may emerge, such as layer anti-ferromagnetic phase and paramagnetic insulator phase. Previous work mainly focus on the electronic properties of BHL^17^,^18^,^19^,^20^,^21^,^22^,^23^,^24^, and the phase diagram for the magnetic phase transition induced by the interaction and dimers are still absent. Moreover, the progress of optical lattice provides us a useful tool to set a controllable and clearness experimental platform to simulate the strongly correlated BHL, in which the interaction between trapped fermionic cold atoms can be tuned by the Feshbach resonance^25^,^26^,^27^,^28^,^29^,^30^,^31^.

The dynamical mean-field theory (DMFT) has been proved to be a very useful and effective tool^32^,^33^,^34^,^35^, which has made significantly progress as in the study of metal-insulator phase transition. The DMFT is exact when investigating the strongly correlated system in the infinite-dimension, in which the self-energy is independent of momentum. However, in low-dimensional systems, the quantum fluctuation and short range correlations play important roles, which are ignored in DMFT. The cellular DMFT (CDMFT), as a cluster extension of DMFT, effectually incorporates the spatial correlations by mapping the many-body problem into finite clusters embedding in a self-consistent media^36^,^37^,^38^,^39^. In two-dimensional systems CDMFT is more precise than DMFT, and is more effective to investigate the phase transition in low and multi-component systems^31^,^40^,^41^.

In this report, we investigate the finite temperature metal-insulator and magnetic phase transition in strongly correlated bilayer honeycomb lattice (BHL) by combining the cellular dynamical mean-field theory (CDMFT) with continue-time quantum Monte Carlo (CTQMC) method^42^. A phase transition from paramagnetic phase to anti-ferromagnetic phase occurs by investigating the magnetization. In a proper value of inter-layer hopping, a novel layer anti-ferromagnetic phase emerges. The layer anti-ferromagnetic phase can be broken by the increasing inter-layer hopping, and a paramagnetic phase can be found. Moreover, the nonlocal inter-layer hopping plays an important role on localizing the free election and modifying the spatial distribution of the electron in lattice sites, especially in dimer sites. For instance, the dimer sites of our system are double occupied, and the non-dimer sites are single occupied. We have also presented the DOS, double occupancy, and fermi surface below, which can be directly detected in future experiments.

## Results

### The strongly correlated bilayer honeycomb lattice

**Figure 1:**
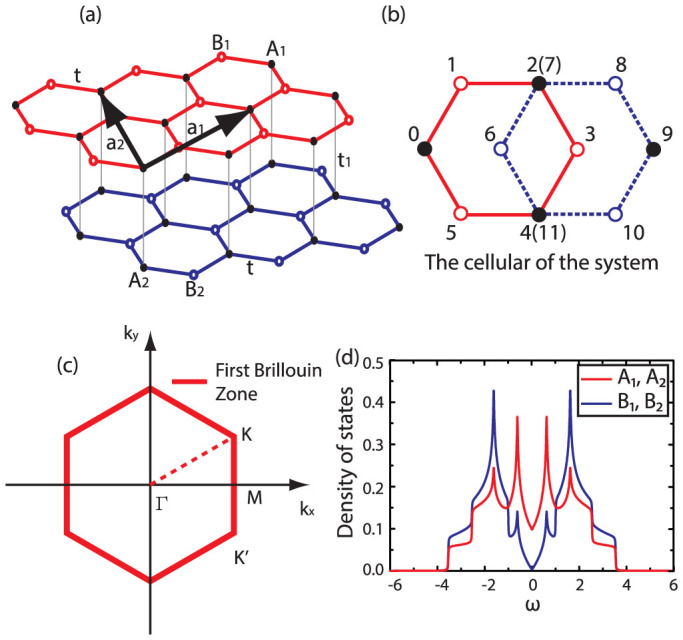
The structure of bilayer honeycomb lattice and its qualities in the non-interacting limit. (a): Bernal stacking of the bilayer honeycomb in real-space with intra- and inter-layer hopping *t* and *t*_1_ between the sublattice *A*_1_, *B*_1_ on top-layer and *A*_2_, *B*_2_ on bottom-layer. The black arrows *a*_1_ and *a*_2_ are the lattice vectors. (b): The cellular of our bilayer system in cellular dynamical mean-field theory (CDMFT). The red solid line containing sites 0, 1, 2, 3, 4, 5 belongs to top-layer and blue dotted line with sites 6, 7, 8, 9, 10, 11 belongs to bottom-layer. (c): Reciprocal lattice of bilayer honeycomb lattice. The thick red line shows the first Brillouin zone. The Γ, *K*, *M* and *K*′ points denote high symmetry points in first Brillouin zone. (d): Density of states of our system for *A*_1_/*A*_2_ and *B*_1_/*B*_2_ sites when *U* = 0.

Fig. 1(a) shows the lattice structure of BHL. *A*_1_ and *B*_1_ denotes the sites on top-layer, while *A*_2_, *B*_2_ signs the bottom-layer sites. The inter-layer band connects *A*_1_ (top) and *A*_2_ (bottom) sites. The first Brillouin zone can be found in Fig. 1(c), in which the Γ, *K*, *M* and *K*′ shows the points with different asymmetry in the *k*-space. The non-interaction density of states (DOS) for different sites when *t*_1_ = *t* is shown in Fig. 1(d), which is different from the mono-layer honeycomb lattice^43^. The low-energy dispersion is quadratic, which is linear in the mono-layer case. There is no band gap between the conduction and valence bands in BHL. In this report, we investigate the phase transition on the half-filling BHL. We consider the standard Hubbard model: 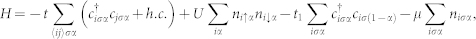
where *t* is the intra-layer nearest-neighbor hopping energy, *U* is the Coulomb interaction, *t*_1_ denotes the inter-layer hopping energy, *µ* is the chemical potential, which is adjusted to keep the system at half-filling, 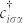 and *c_iσα_* denote the creation and annihilation operators of fermionic atoms on site *i* with spin *σ* respectively, and *α* shows the layer parameter (*α* = 0 means the top-layer, and *α* = 1 denotes the bottom-layer), 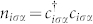 corresponds to the density operator. Here we set *t* = 1, which is also used as the energy unit in this report.

### The metal-insulator phase transition

**Figure 2:**
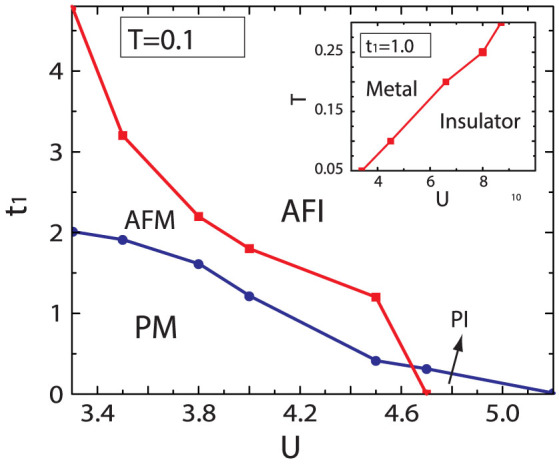
Metal-insulator phase diagram in fixed *T* or *t*_1_. Phase diagram as a function of inter-layer hopping *t*_1_ and interaction *U* at *T* = 0.1. The red solid line shows the boundary of metal-insulator phase transition, and the blue solid line denotes the magnetic phase transition, which divides the phase into paramagnetic metal (PM: *M* = 0 and Δ*E* = 0), paramagnetic insulator (PI: *M* = 0 and Δ*E* ≠ 0), anti-ferromagnetic metal (AFM: *M* ≠ 0 and Δ*E* = 0) and anti-ferromagnetic insulator (AFI: *M* ≠ 0 and Δ*E* ≠ 0). Inset: Phase diagram as a function of temperature *T* and interaction *U* at fixed *t*_1_.

The *t*_1_ − *U* phase diagram is shown in Fig. 2. The phase boundary of the metal and insulator is shown by the red solid line with square plots. When *t*_1_ = 0 the critical point of metal-insulator phase transition is *U_c_* = 4.7, meaning that BHL degenerates into a mono-layer one. This result is the same as Ref. 16. In order to study the magnetic phase transition, we define the staggered magnetization as *M* = Σ*_i_* *sgn*(*i*)(< *n_i_*_↑_ > − < *n_i_*_↓_ >), where *i* denotes the lattice index, *sgn*(*i*) = 1 for *A*_1_ and *A*_2_ sites and *sgn*(*i*) = −1 for *B*_1_ and *B*_2_ sites. The phase boundary of non-magnetic state and magnetic state is shown by blue solid lines with circle points. As shown in Fig. 2, when 2 < *t*_1_ < 4.8, anti-ferromagnet can be found. When *U* is increased, a phase transition from gapless anti-ferromagnetic metal (AFM), where Δ*E* = 0, *M* ≠ 0 and Δ*E* here is single particle gap, to gapped anti-ferromagnetic insulator (AFI), where Δ*E* ≠ 0, *M* ≠ 0, occurs. When *t*_1_ < 2, a paramagnetic metal (PM), where Δ*E* = 0, *M* = 0, is formed, and a PM-AFM-AFI transition happens when *U* is increased. In the value of 0 < *t*_1_ < 0.2, when *U* > 4.7, a small region, which is named as paramagnetic insulator (PI) is found with Δ*E* ≠ 0 and *M* = 0.

**Figure 3:**
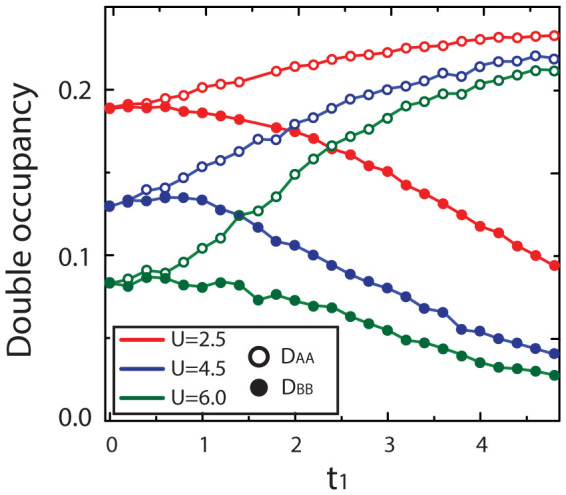
The evolution of double occupancy *D_occ_*. The double occupancy as a function of inter-layer hopping *t*_1_ for different interaction *U* at temperature *T* = 0.1. The dimer sites tend to be double occupied however non-dimer sites tend to be single occupied, with increasing *t*_1_.

Double occupancy is always used to measure the localization of the electrons directly, which is an important parameter to observe the metal-insulator phase transition^44^. In this report, we investigate the double occupancy 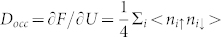 as a function of inter-layer hopping *t*_1_ for various interaction *U* [see in Fig. 3], where *F* is the free energy. We use *D_AA_* to describe the *D_occ_* for *A*_1_ and *A*_2_ sites (hollow circles in Fig. 3), and *D_BB_* the *D_occ_* for *B*_1_ and *B*_2_ sites (solid circles in Fig. 3). When *t*_1_ = 0, *D_occ_* decrease when *U* is increased showing the localization is enforced by the interaction. When *t*_1_ ≠ *t*, *D_AA_* is different with *D_BB_*. It is found that when *t*_1_ is increased, *D_AA_* increases while *D_BB_* decreases. This suggests the itinerancy of electrons in dimer sites is enhanced due to the increasing inter-layer hopping. However, be different with *D_AA_*, *D_BB_* decreases while *t*_1_ is increased. These results suggest the intra-hopping between dimer and non-dimer sites is weaken due to the forming of spin-polarized electrons in dimer sites.

**Figure 4:**
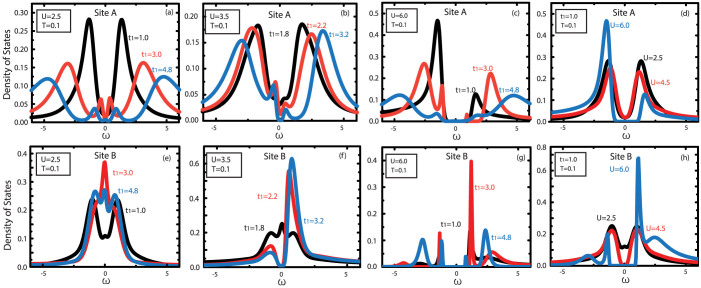
The density of states. (a) – (c), (e) – (g): The density of states as a function of frequency *ω* for different inter-layer hopping *t*_1_ at temperature *T* = 0.1. (a), (e): The metallic phase at *U* = 2.5 for A sites and B sites. (b): For A sites, as we increase *t*_1_, system undergoes a phase transition from metal (at *U* = 3.5 and *t*_1_ = 1.8) to insulator (at *t*_1_ = 3.2). Single particle excitation gap opens at about *t*_1_ = 3.2. (f): For B sites, same tendency occurs and the critical point is also at about *t*_1_ = 3.2. (c), (g): The insulating phase at *U* = 6.0. There is a visible single particle excitation gap around the Fermi energy. (d), (h): The density of states as a function of *ω* for different *U* with fixed *t*_1_.

We derive DOS from the imaginary time Green's function *G*(*τ*) to observe the single particle spectral for different interaction *U* and *t*_1_ by the maximum entropy method^45^,^46^. Fig. 4(a) and Fig. 4(e) shows the DOS for different inter-layer hopping when *U* = 2.5, *T* = 0.1 for A sites and B sites. It is found that, in weak interaction, the inter-layer hopping *t*_1_ does not affect the metallic properties of BHL. In Fig. 4(b) and Fig. 4(f), we can find that the system keeps at a metallic state when *t*_1_ = 1.8, and an obvious pseudo-gap is found when *t*_1_ = 2.2. A metal-insulator transition happens when *t*_1_ = 3.2 for *U* = 3.5 and *T* = 0.1 for both A and B sites. When interaction being large, such as *U* = 6.0 [see Fig. 4(c) and Fig. 4(g)], the system stays at an insulating state, which is insensitive of *t*_1_. Fig. 4(d) and Fig. 4(h) show the DOS for different *U* when *t*_1_ = 1.0 and *T* = 0.1. An opened gap is found when the interaction is increased, which indicates a phase transition from metal to insulator. These results mean that the inter-layer hopping and Coulomb interaction play same roles for the metal-insulator phase transition.

**Figure 5:**
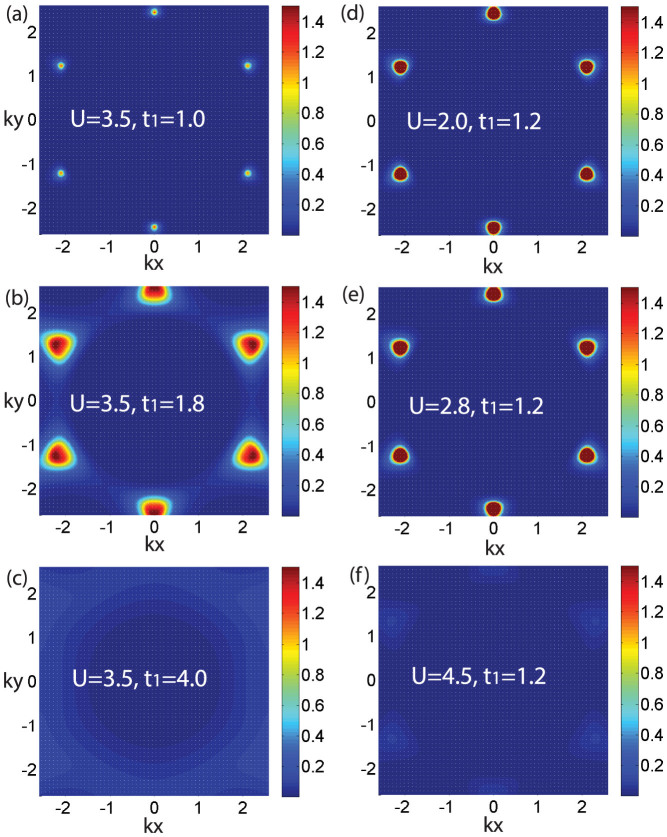
The evolution of spectral function near Fermi surface. The spectral function *A*(*k*, *ω* = 0) near the Fermi surface for different inter-layer hopping *t*_1_ at *T* = 0.1 and *U* = 3.5: (a) *t*_1_ = 1.0, (b) *t*_1_ = 1.8, (c) *t*_1_ = 4.0. The *A*(*k*, *ω* = 0) when *T* = 0.1 and *t*_1_ = 1.2 for different interaction *U*: (d) *U* = 2.0, (e) *U* = 2.8, (f) *U* = 4.5.

In this report, the Fig. 5 shows the spectral function *A*(*k*, *ω* = 0) near the Fermi surface for various *U* and *t*_1_. *A*(*k*, *ω*) is defined as: 
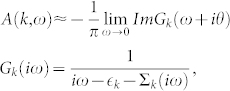
where *iω* is the Fermionic Matsubara frequency, 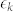 denotes the dispersion relation, Σ*_k_*(*iω*) corresponds to the *k*-dependent self-energy, *k* is the wave vector in the first Brillouin zone. Figs. 5(a)–(c) show the *A*(*k*, *ω* = 0) for different *t*_1_ when *U* = 3.5. When *t*_1_ < 3.2, we can find that the peaks of *A*(*k*, *ω* = 0) distribute in the *K* and *K*′ points [see Fig. 1(c)] in *k*-space, displaying a metallic behavior [see Figs. 5(a), (b)]. When *t*_1_ = 4.0, the *A*(*k*, *ω* = 0) ≈ 0 [see Fig. 5(c)], meaning that a gap can be found near the Fermi energy. This gapped behavior shows the system is an insulator. Similar behavior is found when *t*_1_ = 1.2 and *U* is increased [see Figs. 5(d) – (f)]. It means both spin-polarization and strongly correlated interaction can induce the localization of electrons, driving the system from metal to insulator.

### The magnetic phase transition and a novel layer anti-ferromagnetic phase

**Figure 6:**
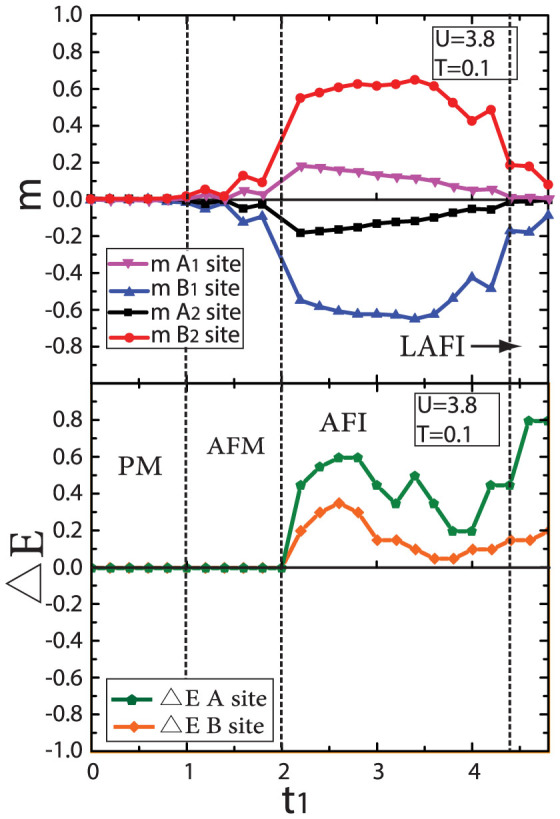
The evolution of the magnetic order parameter *m*. The evolution of magnetic order parameter *m* at *T* = 0.1 and *U* = 3.8. A paramagnetic phase is found in weak *t*_1_. For *t*_1_ > 1.0, the magnetic parameter *m* is nonzero and has opposite sign between *A*_1_/*A*_2_ sites and *B*_1_/*B*_2_ sites. The system goes into anti-ferromagnetic phase. At large *t*_1_ the magnetization of *A*_1_/*A*_2_ sites are more easily decreasing to zero while *B*_1_/*B*_2_ sites are still nonzero. The system is layer anti-ferromagnetic phase. Single particle excitation gap ΔE denoted by the dark green solid line and orange solid line, divide the phase into paramagnetic metal (PM), anti-ferromagnetic metal (AFM), anti-ferromagnetic insulator (AFI) and layer anti-ferromagnetic insulator (LAFI).

**Figure 7:**
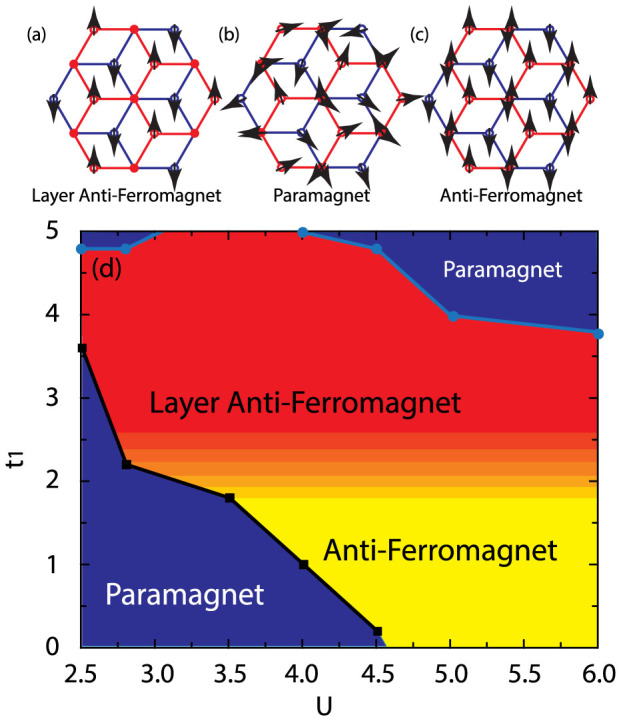
The phase diagram of magnetic phase transition. In weak interaction *U* and weak inter-layer hopping *t*_1_, the system is paramagnet. When we increase *U*, the system undergoes a magnetic phase transition to anti-ferromagnet. When we increase *t*_1_, the magnetization of *A*_1_/*A*_2_ sites decrease to zero while *B*_1_/*B*_2_ sites stay finite. The system becomes layer anti-ferromagnet. In the region where *t*_1_ > 5.0, the system returns to paramagnetic phase.

In strongly correlated BHL, charge imbalance between the two sublattices sites causes different kinds of magnetic spontaneous symmetry breaking, dividing the sites into dimer sites and non-dimer sites. In order to study the magnetic order, we use a magnetic order parameter defined as 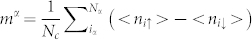, where *α* denotes the sublattice *A*_1_, *A*_2_, *B*_1_ and *B*_2_, *i_α_* is the lattice index for sublattice *α*, and *N_α_* means the site number for sublattice *α* [see in Fig. 1(a) and Fig. 1(b)]. Parameter < *n_iσ_* > indicates the electron density in lattice site *i* with spin index *σ* (we set magnetization of *A*_1_ as positive sign). Fig. 6 shows the evolution of *m^α^* as a function of *t*_1_ for *U* = 3.8. It is found that when *U* = 3.8, *m* is zero for *t*_1_ < 1.0, denoting that no magnetic order is formed for weak *t*_1_. A magnetic state with anti-ferromagnetic order is found when *t*_1_ > 1.0. When *t*_1_ > 4.4, the 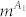 and 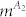 decrease to zero, while 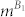, 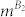 are still finite, meaning a phase with non-magnetic order and magnetic order coexisting. This novel phase is called layer anti-ferromagnetic state, in which the dimer sites are non-magnetic and non-dimer sites are magnetic. The sketches of the possible magnetic order existing in BHL is shown in Figs. 7(a) – (c).

Finally, the phase diagram of magnetization about *t*_1_ and *U* for *T* = 0.1 is shown in Fig. 7. A phase transition from paramagnet to anti-ferromagnet is found when *U* is increased. A layer anti-ferromagnetic phase is found for large *t*_1_, in which the magnetic of dimer sites keeps zero while non-dimer sites is nonzero. When *U* > 3.7, a phase transition from layer anti-ferromagnetic state to paramagnetic state is found.

### Experimental protocol

We propose an experiment setup to investigate the phase transition in strongly correlated bilayer honeycomb lattice (BHL). The ^40^K atoms can be produced as a pure fermion condensate by evaporative cooling^47^, which provides two hyperfine states |*F*,*m_F_*〉 = |9/2, −9/2〉 ≡ | ↑〉 and |*F*,*m_F_*〉 = |9/2, −7/2〉 ≡ | ↑〉^48^. Three standing-wave laser beams are used to form the honeycomb lattice, and two extra laser beams along the *z* direction suppress the tunneling between layers^28^. The potential of optical lattice is given by *V_h_*(*x*, *y*) = *V*_0_Σ*_j_*_ = 1,2,3_ *sin*^2^[*k*(*x* cos *θ_j_*+*y* sin *θ_j_*)+*π*/2], where *θ*_1_ = *π*/3, *θ*_2_ = 2*π*/3, *θ*_3_ = 0. Then, we use another three standing-wave laser beams with a 2*π*/3 angle between each other to form triangular lattice. The potential is given by 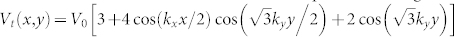. *k_x_* and *k_y_* are the two components of the wave vector *k* = 2*π*/*λ* in these two types of lattices, where *λ* = 738 *nm* is wavelength of the laser, and *V*_0_ is given in recoil energy 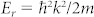. Inserting the triangular lattice between two layers of honeycomb lattice, the Bernal stacking BHL with trapped ^40^K atoms can be formed^49^,^50^. In BHL the intra-layer hopping 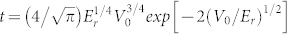 is adjusted by the periodic potential of laser beam and *t*_1_ can be tuned by changing the wavelength of laser beam in *z* direction. The on-site interaction 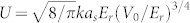 is determined by the s-wave scattering length *a_s_*, which can be tuned by Feshbach resonance. The temperature can be extracted from the time-of-flight images^51^.

It should be mentioned that, we can detect the numbers of double occupied sites to confirm whether the metal-Mott insulator transition happens. Firstly, we have to increase the depth of the optical lattice to prevent further tunneling of atoms. Next, the energy of the atoms on doubly occupied sites is shifted by approaching a Feshbach resonance. Then the one spin component of atoms on double occupied sites is transferred to a new magnetic sublevel by radio-frequency pulse method. Finally, the double occupancy can be deduced by the absorption images^52^,^53^.

To get Fermi surface in experimental, we ramp down the optical lattice slowly enough first, and the atoms stay adiabatically in the lowest band while quasi-momentum is approximately conserved. Then the lattice potential is lowered to zero rapidly, by switching off the confining potential and the atoms can expand ballistically for several milliseconds. The Fermi surface can be obtained by a absorption image^54^,^55^.

## Discussion

In this work, we have investigated the metal-insulator transition and magnetic phase transition in strongly correlated bilayer honeycomb lattice using cellular dynamical mean-field theory (CDMFT) combining with continue-time quantum Monte Carlo (CTQMC) method. In lower temperature case we map the phase diagram as a function of interaction *U*, inter-layer hopping *t*_1_ and magnetization *m*. It shows that the inter-layer hopping affects the electrons to form spin-polarized electrons, and an insulating state is induced. A layer anti-ferromagnetic phase is found at large *t*_1_, in which the magnetization of dimer sites is zero while non-dimer keeps finite value. Therefore, the inter-layer hopping *t*_1_ plays an important role to form a singular magnetic spontaneous symmetry breaking phase. Our study may provide a helpful step for understanding the interaction and inter-layer hopping driven metal-insulator transition, the exotic magnetic order with asymmetry breaking and the possible magnetic-nonmagnetic order coexisting state.

## Methods

### The cellular dynamical mean-field theory

We combine the cellular dynamical mean-field theory (CDMFT) with continuous time quantum Monte Carlo (CTQMC) method to determine the metal-insulator transition and magnetic phase transition in the strongly correlated bilayer honeycomb lattice. In low-dimensional systems, quantum fluctuations are much stronger than the higher dimensions. The nonlocal effect is much important in this case. Dynamical mean-field theory ignoring the nonlocal correlations leads lots of errors in calculation. Therefore, we use CDMFT, as the advanced method in our work. We map the original lattice onto a 12-site effective cluster embedded in a self-consistent bath field [see Fig. 1(b)]. Starting with a guessing self-energy Σ(*iω*) (which is independent of momentum^56^), we can get the Weiss field *G*_0_(*iω*) obtained by the coarse-grained Dyson equation: 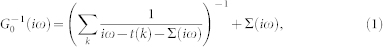
where *iω* is Fermionic Matsubara frequency, *µ* is the chemical potential, *k* is in the reduced Brillouin zone of the super-lattice, and *t*(*k*) is hopping matrix for the super-lattice. The form of *t*(*k*) is: 
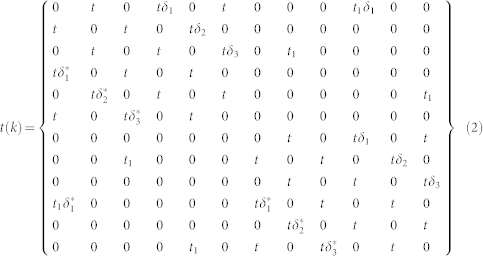
where 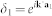, 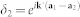, 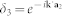 and **a**_1_, **a**_2_ are real lattice vectors as shown in Fig. 1(a). The cluster Green's function *G*(*iω*) can be gotten by the impurity solver. In our work, we use the numerically exact CTQMC simulation as impurity solver and take 5 × 10^6^ QMC sweeps for each CDMFT loop^42^. The new self-energy Σ(*iω*) is recalculated by the Dyson equation: 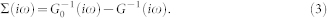
This iterative loop repeated until self-energy is converged.

The CTQMC method as impurity solver can be taken as follows. We start the procedure at partition function, which can be written as: 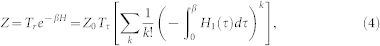
where *T_τ_* is time-ordering operator, 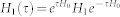 is *H*_1_ in the interaction picture, and 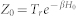 is a partition function for the unperturbed term. Putting *H*_1_ = *U* Σ*_i_n_i_*_↓_*n_i_*_↑_ in Eq. 4, the partition function is 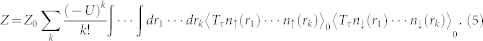
Here 〈〉_0_ indicates a theromdynamic average with respect to 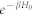. Using Wick's theorem, for each order in *k*, 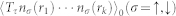 can be written as determinant *detD*(*k*): 
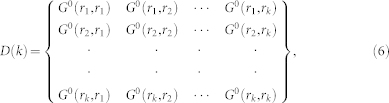
where *G*^0^ is non-interacting Green's function. There is no spin index in *D*(*k*) for the determinants of spin-un and -down being equivalent. Like classical Monte Carlo, by integrand of Eq. 5, we can get the weight of order *k*

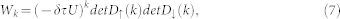
where *δτ* = *β*/*L* is slice of imaginary time. We can get the standard Metropolis acceptance ratio *R* of adding vertex by the detailed balance condition: 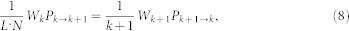

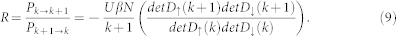


Here *P_k_*_→*k*+1_ is the probability to increase the order from *k* to *k* + 1 (*P_k_*_+1→*k*_ the probability to decrease the order from *k* + 1 to *k*), 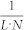 is probability to choose a position in time and space for vertex you intend to add while 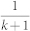 is the probability to choose one vertex you intend to remove of from the existing *k* + 1 noes. To calculate the ratio *R*, we have to deal with the function *detD*(*k* + 1)/*detD*(*k*). 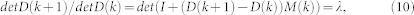

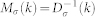
, we can easily get the value of *λ* in matrix form: 
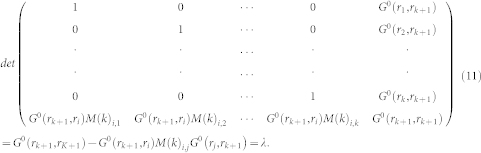
Then it is easy to obtain the update *M* for the order *k* + 1 by numerical method: 
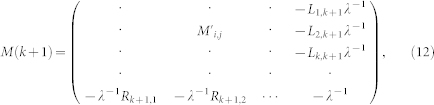
where the factor of the matrix is 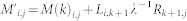, *R_i_*_,*j*_ = *G*^0^(*i*, *l*)*M*(*k*)*_l_*_,*j*_ and *L_i_*_,*j*_ = *M*(*k*)*_i_*_,*l*_*G*^0^(*l*, *j*). For the step *k* − 1, we can also get the radio *R* and update formulas of *M*(*k* − 1): 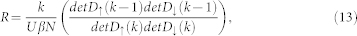

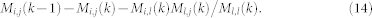


Using the update formula for *M*, the Green's function can be obtained both in imaginary time and at Matsubara frequencies: 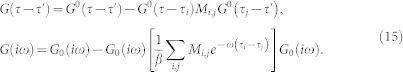
Here *G*_0_(*iω*) is a bare Green's function.

## Author Contributions

H.S.T. performed calculations. H.S.T., Y.H.C., H.D.L., H.F.L., W.M.L. analyzed numerical results. H.S.T., Y.H.C., W.M.L. contributed in completing the paper.
